# Toward a forest biomass reference measurement system for remote sensing applications

**DOI:** 10.1111/gcb.16497

**Published:** 2022-11-08

**Authors:** Nicolas Labrière, Stuart J. Davies, Mathias I. Disney, Laura I. Duncanson, Martin Herold, Simon L. Lewis, Oliver L. Phillips, Shaun Quegan, Sassan S. Saatchi, Dmitry G. Schepaschenko, Klaus Scipal, Plinio Sist, Jérôme Chave

**Affiliations:** ^1^ Evolution and Biological Diversity (EDB) CNRS/IRD/UPS Toulouse France; ^2^ Forest Global Earth Observatory Smithsonian Tropical Research Institute Washington District of Columbia USA; ^3^ Department of Geography University College London (UCL) London UK; ^4^ NERC National Centre for Earth Observation (NCEO) London UK; ^5^ Department of Geographical Sciences University of Maryland College Park Maryland USA; ^6^ GFZ German Research Centre for Geosciences Potsdam Brandenburg Germany; ^7^ School of Geography University of Leeds Leeds UK; ^8^ School of Mathematics and Statistics University of Sheffield Sheffield UK; ^9^ Jet Propulsion Laboratory (JPL) California Institute of Technology Pasadena California USA; ^10^ International Institute for Applied Systems Analysis (IIASA) Laxenburg Austria; ^11^ Center for Forest Ecology and Productivity of the Russian Academy of Sciences Moscow Russia; ^12^ European Space Agency (ESA) Frascati Italy; ^13^ Forests and Societies CIRAD Montpellier France

**Keywords:** aboveground biomass, carbon, Earth Observation, forest vegetation, permanent plots, representativeness, validation

## Abstract

Forests contribute to climate change mitigation through carbon storage and uptake, but the extent to which this carbon pool varies in space and time is still poorly known. Several Earth Observation missions have been specifically designed to address this issue, for example, NASA's GEDI, NASA‐ISRO's NISAR and ESA's BIOMASS. Yet, all these missions' products require independent and consistent validation. A permanent, global, in situ, site‐based forest biomass reference measurement system relying on ground data of the highest possible quality is therefore needed. Here, we have assembled a list of almost 200 high‐quality sites through an in‐depth review of the literature and expert knowledge. In this study, we explore how representative these sites are in terms of their coverage of environmental conditions, geographical space and biomass‐related forest structure, compared to those experienced by forests worldwide. This work also aims at identifying which sites are the most representative, and where to invest to improve the representativeness of the proposed system. We show that the environmental coverage of the system does not seem to improve after at least the 175 most representative sites are included, but geographical and structural coverages continue to improve as more sites are added. We highlight the areas of poor environmental, geographical, or structural coverage, including, but not limited to, Canada, the western half of the USA, Mexico, Patagonia, Angola, Zambia, eastern Russia, and tropical and subtropical highlands (e.g. in Colombia, the Himalayas, Borneo, Papua). For the proposed system to succeed, we stress that (1) data must be collected and processed applying the same standards across all countries and continents; (2) system establishment and management must be inclusive and equitable, with careful consideration of working conditions; and (3) training and site partner involvement in downstream activities should be mandatory.

## Graphical abstract

A permanent, global, in situ, site‐based forest biomass reference measurement system relying on ground data of the highest possible quality is needed to validate products from biomass‐related Earth Observation missions. Here, we have assembled a list of almost 200 high‐quality sites. We explore how representative these sites are in terms of their coverage of environmental conditions, geographical space and biomass‐related forest structure, compared to those experienced by forests worldwide.

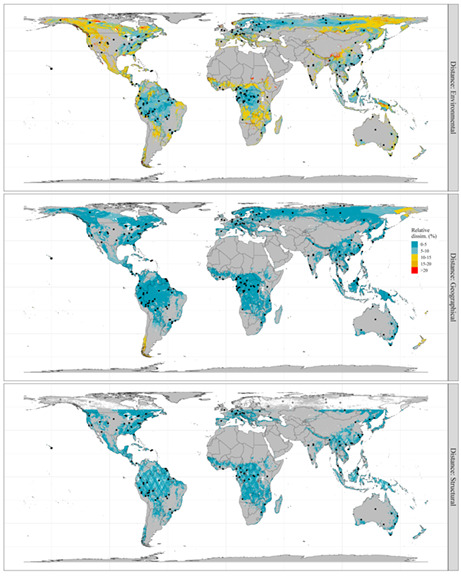

## 1 INTRODUCTION

Plants store about 80% of the Earth's biomass carbon (Bar‐On et al., 2018), with forests constituting by far the largest plant carbon pool (ca. 80%; Pan et al., 2013). However, estimates of the spatial distribution and temporal variation of this carbon pool are still imprecise (Harris et al., 2021; Santoro et al., 2021). While forests are vulnerable to global change (Brienen et al., 2020; McDowell et al., 2020; Schimel et al., 2015), they currently provide a carbon sink (e.g. Pan et al., 2011; van Marle et al., 2022) and could contribute further to mitigating climate change given the large potential of intact and regenerating forests for carbon uptake and storage (Chazdon et al., 2016; Requena Suarez et al., 2019). Understanding the nature and distribution of forest carbon fluxes due to land use change and other processes depends critically on mapping the current distribution of vegetation biomass. Moreover, a key factor in projecting how and where forest regeneration or restoration projects would be most effective is detailed, spatially explicit knowledge of local biomass storage potential (see e.g. Heinrich et al., 2021).

The remote sensing community has made substantial investments to address the global challenge of mapping forest carbon stores, fluxes and their sequestration potential. Several ongoing and upcoming Earth Observation (EO) missions are designed to measure key structural parameters of the world's forests, their carbon stores and their carbon fluxes, for example, NASA's GEDI (Dubayah et al., 2020), NASA‐ISRO's NISAR (NISAR, 2018) and ESA's BIOMASS (Quegan et al., 2019). Each is expected to deliver biomass maps with associated uncertainty. Their coverage, spatial resolution and range depend on mission specifications (e.g. coverage of Earth's surface between 51.6° N and 51.6° S for GEDI, biomass up to 100 Mg/ha for NISAR). Although these missions offer novel approaches to mapping forest carbon, their products require validation using standard procedures to bolster their uptake for a broad range of uses, including climate modelling, national reporting and land use management (Duncanson et al., 2019). Only if the accuracy and uncertainty of biomass maps are comprehensively assessed and quantified will they meet the needs of the user communities.

How should this be done? We argue that given the wide range of users, instrument sensors, platforms, often limited lifetimes and pace of technological change, validation strategies need a clear long‐term ground vision. This means developing a consistent approach that covers the world's forests and is built to last. It requires designing and maintaining a permanent, global, in situ, site‐based forest biomass reference measurement (henceforth, FBRM) system to enable independent validation of biomass products and proper quantification of associated uncertainty. Building and sustaining this high‐quality distributed system of FBRM sites needs to be an integral part of all EO missions aimed at mapping forest biomass.

In compliance with the good practices protocol for the validation of aboveground woody biomass products (Duncanson et al., 2021), the design of the FBRM system needs to follow a number of principles: (1) ground data should be of the highest possible quality, with large permanent sampling plots (at least 1 ha in size, 10 ha minimum in total), and airborne LiDAR coverage (at least 1000 ha) plus complementary terrestrial LiDAR acquisitions. The procedures for data acquisition and database compilation should be standardized by following established protocols, and all data should be collected as synchronously as possible with EO measurements; (2) the system should cover the broadest possible range of environmental, geographical and structural conditions, so as to maximize the robustness of validation activities; (3) the selection of sites should be pragmatic, that is, focusing on sites where previous expertise and capacity have been built and future operation is highly likely. Establishing and maintaining multiple, high‐quality permanent plots is challenging, especially in the tropics (Davies et al., 2021; ForestPlots.net et al., 2021). Therefore, it is strategically sensible while building a potential FBRM system to leverage the experience, knowledge and investment of all stakeholders engaged in long‐term permanent plot networks, from data originators (e.g. forest workers) to data curators. And for any such system to be fair and sustainable, the needs of data contributors should be of pivotal concern (de Lima et al., 2022).

Previous experience with the validation of EO products demonstrates the value of highly integrated FBRM sites compared to widely distributed small forest samples as established by most national forest inventories. This is because validation of EO‐derived biomass maps depends strongly on accurate spatial registration of the ground plots, and because biomass estimates from individual plots are informative for calibration/validation only if the plots are large enough (Réjou‐Méchain et al., 2014). All the aforementioned conditions for the inclusion of sites in a global monitoring system are difficult to meet, and for the moment, validation efforts for each individual EO mission have been based on a handful of sites.

How many observation sites would be necessary for global validation of biomass maps, and where should they be located? From a validation perspective, these sites should ideally span a wide range of biomass, and should encompass a variety of forest structures for any given level of biomass. But from an ecological point of view, the sites should cover an extensive range of bioclimatic and biogeographic conditions, as well as contrasting topographies, soil types and geological substrates, and be exposed to varying levels and types of anthropogenic pressures or natural disturbances. Given the enormous extent and diversity of forests globally, the replication of high‐quality observation sites at thousands of locations is unrealistic, so the theoretical challenge in allocating limited resources to locations involves maximizing their distance from each other along key dimensions, to ensure an optimized coverage of conditions experienced by forests around the world. However, because these sites should ideally already be established (Chave et al., 2019), the problem of site selection is constrained by what is available. Here, we have assembled a list of almost 200 potential FBRM sites through an in‐depth review of the literature and expert knowledge. The aim of this study is to evaluate how representative these sites are in terms of their coverage of three key biomass‐related dimensions, that is, environmental, geographical and structural, in the context of forests worldwide.

Specifically, we ask the following research questions: (1) how well does a selection of existing forest sites represent environmental conditions, geographical space and forest structure globally?; (2) which combination of sites best represents each of the three biomass‐related dimensions over global forested areas, for any given number of sites?; (3) how does a combination of potential FBRM sites compare in terms of representativeness with an equivalent number of forested locations randomly selected over the globe?; (4) where should efforts be invested to improve the environmental, geographical and structural coverage of the proposed FBRM system, possibly going beyond existing plots?

## 2 MATERIALS AND METHODS

### 2.1 Potential FBRM sites

We assembled a list of sites meeting all or most of the quality criteria required to become part of the FBRM system (e.g. plot size, likeliness to be revisited). We screened the following continental to global‐scale forest plot networks for potential sites of interest: AfriTRON (Hubau et al., 2020), ForestGEO (Davies et al., 2021), IIASA (Schepaschenko et al., 2017), NEON (Metzger et al., 2019), RAINFOR (ForestPlots.net et al., 2021), SEOSAW (The SEOSAW Partnership, 2020), TERN (Cleverly et al., 2019) and TmFO (Sist et al., 2015). Peer‐reviewed and grey literature were also searched, and expert knowledge mobilized through consultation with key stakeholders, such as EO mission research scientists, space agencies and national forest/forestry departments. We tried to be as thorough and exhaustive as possible but some high‐quality plots and networks might have escaped our notice and readers are encouraged to contact the corresponding author to notify us of this.

The screening resulted in a list of 195 potential FBRM sites (Table S1). Among these, plot cumulative area ranged from 0.5 ha for several of the Siberian sites to 125 ha at Paracou, French Guiana. About two‐thirds of the sites had a plot cumulative area ≥10 ha (*n* = 132), with about half of those that did not located in the Palearctic (*n* = 30). Potential FBRM sites were present in every forested biome, sensu Whittaker (1975), yet the coverage of annual precipitation and mean temperature gradients was uneven (Figure S1). About three‐quarters were affiliated to (at least) one of the eight large‐scale networks. The rest were usually monitored by research institutes, universities, or national forest/forestry departments.

We use the terminology of ‘potential’ FBRM sites, mindful that this list is likely to change in the future for various reasons. One is that most of the sites have not formally agreed to join the proposed system of FBRM sites (and many have probably not heard about the concept yet). Plus, some sites may in the end prove unsuitable, and others may join the initiative. However, the fairly large sample of sites represented in the list reported here is a useful step to test this study's research questions.

### 2.2 Geographical information and study area

All spatial data were reprojected using a global equal‐area map projection to reflect the respective and relative area contributions of realms and continents. EASE‐Grid 2.0 (epsg:6933), version 2 of the Equal‐Area Scalable Earth Grid (Brodzik et al., 2012), is commonly used for satellite‐based data distribution (see e.g. GEDI; Dubayah et al., 2021). This projection is preferable to the longitude–latitude coordinate reference system (epsg:4326), that is neither equal area nor conformal. The coarsest spatial resolution of all spatial datasets used in this study (2.5 arc‐min, which is about 5 km at the equator, for the TerraClimate dataset; Abatzoglou et al., 2018) was chosen, and all datasets were resampled accordingly. Following reprojection and resampling, gridded data were generated over 2920 rows and 6940 columns, that is 20,264,800 cells in total.

To restrict our analysis to forests, we built a forest mask using land cover data for 2020 from the ESA CCI Land Cover project. The original dataset (300 m spatial resolution; epsg:4326) was reprojected and resampled to 5 km (mode retained). The mask included cells with tree‐dominated land cover classes (see Supporting Information for more details), for a total of 1,728,368 cells (i.e., around 43 million km^2^). Non‐tree‐dominated land cover classes such as shrubland, grassland and cropland are also pools of carbon, but were not considered here.

### 2.3 Environmental space

Climatic, topographic and edaphic variables are widely used to investigate the influence of the environmental space on forest structure, composition and functioning (see e.g. Anderson‐Teixeira et al., 2015; Sullivan et al., 2020).

Temperature and precipitation are key climatic factors influencing vegetation patterns (Holdridge, 1947; Whittaker, 1975), together with their seasonality (Mucina, 2019). So is solar radiation (Cox et al., 2016). Annual mean temperature (°C), temperature seasonality (% coefficient of variation [CV]), annual precipitation (mm), precipitation seasonality (% CV) and solar radiation (W m^−2^) were therefore selected for subsequent analysis. Data were taken from the TerraClimate dataset (original spatial resolution 5 km; Abatzoglou et al., 2018) directly, or could be computed from it following O'Donnell and Ignizio (2012).

Topographic variables and especially elevation also shape the spatial distribution of species and habitats (see altitudinal zonation; von Humboldt & Bonpland, 1805). Data on elevation above sea level (m) were obtained from the EarthEnv project (http://www.earthenv.org/; Amatulli et al., 2018).

Soil physicochemical properties have a direct influence on vegetation, as they partly determine water and nutrient availability (Hulshof & Spasojevic, 2020). Estimated edaphic data were obtained from SoilGrids 2.0 (original spatial resolution 250 m; Poggio et al., 2021). Depth‐weighted averaged values over the three topmost soil layers (i.e. 0–5, 5–15 and 15–30 cm) were computed for each of the 11 variables provided. As in Sullivan et al. (2020), we selected variables representing both soil physical (‘texture’) and chemical (‘fertility’) properties. More specifically, we retained coarse fragment content (% volume), sand fraction (% mass), cation exchange capacity (cmol kg^−1^) and pH (H_2_O) (unitless). We favoured sand fraction over clay fraction (commonly retained in similar analyses), as the latter was modelled less accurately (Poggio et al., 2021).

Some edaphic variables were found to be strongly correlated with climatic ones, like cation exchange capacity and annual mean temperature (Spearman's rank correlation coefficient *ρ* < −.75). This may be because edaphic variables are modelled using other variables, including climatic ones (Poggio et al., 2021). Despite some strong pairwise correlations between the 10 variables selected (5 climatic, 1 topographic and 4 edaphic), we kept them all as indicators of the environmental space as each bears relevant information. Correlation is unlikely to distort results from the analysis of network representativeness described below. Previous studies, for example, Anderson‐Teixeira et al. (2015) or Hoffman et al. (2013), ran the same analysis using an even bigger number of variables (*n* = 17 and *n* = 37, respectively) without considering correlation.

### 2.4 Geographical space

We also explored whether the potential sites were sufficiently distant from each other to cover the entire forested area of the world. Since floristic composition varies greatly across continents, maximizing geographical distance across sites and minimizing the occurrence of geographical gaps is desirable in the optimal design of a reference measurement system.

### 2.5 Structural space

Canopy height and tree cover (TC) fraction are two structural variables commonly used to describe forest structure. Both can be estimated by spaceborne instruments. Canopy height (*H*) information was obtained from the GEDI L3 Gridded Land Surface Metrics, Version 2 dataset (Dubayah et al., 2021). Gridded data at 1 km spatial resolution (mean RH100, i.e. the 100th percentile of waveform energy relative to the ground, computed from individual waveforms collected between April 18th 2019 and April 14th 2021) were averaged to 5 km. We kept 5 km cells only when at least half of their area overlapped with non‐empty 1 km cells. Due to GEDI discrete sampling and ISS‐orbit limited spatial coverage (±51.6° latitude), only about 60% of the potential FBRM sites (*n* = 118) and half of the forested cells (*n* = 829,256) had canopy height information available from GEDI first 2 years of data collection.

Tree cover fraction was also used, based on the PROBA‐V satellite acquisitions for 2019. These data were obtained from Version 3.0.1 of the global land cover maps distributed by the Copernicus Global Land Service (Buchhorn et al., 2020). Original data at 100 m spatial resolution were reprojected and averaged to 5 km.

### 2.6 Analysis of network representativeness

To assess how well a network of observation sites represents environmental, geographical and structural conditions of forested areas globally, we performed a point‐based ‘representativeness of network’ analysis (Anderson‐Teixeira et al., 2015; Hoffman et al., 2013). The principle of this analysis is as follows.

For each site, distances were computed between values at that site and those at any cell of the map included in the forest mask. More precisely, we computed Euclidean distances on standardized variables (after *z*‐score normalization) for the environmental and structural spaces, and great‐circle distance (i.e. the shortest distance between two points on the Earth surface, represented here by a sphere) computed using the haversine formula for the geographical space. This resulted in site‐specific environmental, geographical and structural distance maps. Site‐specific maps referring to the same space were then stacked, and the minimum value retained for each cell to produce environmental, geographical and structural dissimilarity maps. Lastly, maximum environmental, geographical and structural distances were searched for (see Supporting Information for more details) and relative dissimilarity was mapped as a percentage of the normalized value.

The representativeness of network analysis was performed for various sets of contributing sites (i.e. those included in the stack from which minimum values were selected) based on the following selection strategies: all potential FBRM sites, only those with a plot cumulative area ≥10 ha, the *n* most representative potential FBRM sites, *n* randomly selected potential FBRM sites, the *n* most representative virtual sites (i.e. cells with no potential FBRM site identified for the time being) over global forested areas, and *n* randomly selected virtual sites (for *n* ranging from 5 to 118 or 195 depending on selection strategy and space).

To identify the most representative FBRM or virtual sites for a given number of sites, *n*, we performed a partitioning around medoids (PAM) analysis. This clustering technique is suited for our purpose as clusters are built around actual objects (the so‐called ‘medoids’, here potential FBRM sites or cells) and not ‘centroids’ as in the k‐means algorithm (Kaufman & Rousseeuw, 1990). Despite often being regarded as deterministic (see e.g. Reynolds et al., 2006), there might be ties in some cases, for example, during medoid selection when choosing between two objects that may give the same reduction in the cost function, that is, the sum of dissimilarities. In this case, selecting one object over the other would depend on the order in which these two objects were presented to the algorithm. To address this problem, we ran the original PAM algorithm a 100 times for each number, *n*, of potential FBRM sites of interest, each time reshuffling the input dataset, and retained the most frequent combination to serve as the *n* most representative sites. For most representative virtual site selection, the ‘fasterPAM’ algorithm was used on a subset of 20,000 cells geographically spanning global forested areas to reduce the computational burden (Schubert & Rousseeuw, 2021).

Finally, we selected potential FBRM sites randomly and ran the representativeness of network analysis. This operation was repeated 200 times, and median relative dissimilarity values retained for each cell of the study area to produce relative dissimilarity maps. The whole process was also performed for virtual sites selected randomly over global forested areas, with only five repetitions in this case because of computational cost. Only for *n* = 100 were random virtual site selection and subsequent representativeness of network analysis repeated 200 times (median retained for each cell of the forest mask), and the difference between random versus most representative site resulting relative dissimilarity maps computed.

All analyses were conducted using the R statistical computing platform (R Core Team, 2021), and mainly packages ‘cluster’ (Maechler et al., 2021), ‘data.table’ (Dowle & Srinivasan, 2021), ‘gdalUtils’ (Greenberg & Mattiuzzi, 2020) and ‘raster’ (Hijmans, 2021).

## 3 RESULTS

### 3.1 Representativeness of a potential FBRM system with all pre‐existing sites currently identified

**FIGURE 1:**
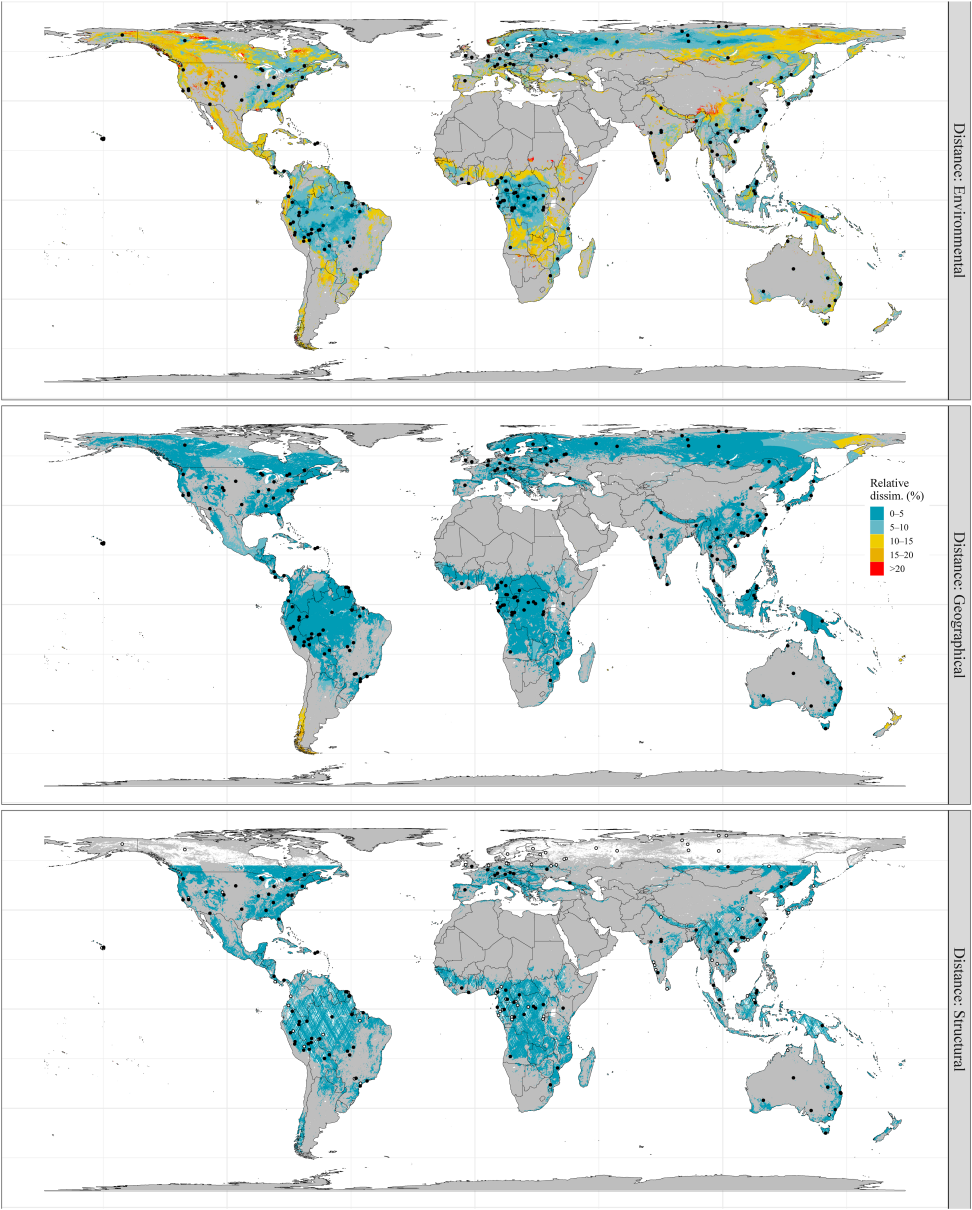
Relative environmental (top), geographical (centre) and structural (bottom) dissimilarities (%) over global forested areas with respect to conditions covered by potential forest biomass reference measurement sites (*n* = 195, top and centre; *n* = 118, bottom). Blank continental areas and hollow points (bottom), respectively, correspond to forested areas and sites not sampled (yet, for those within ±51.6° latitude) by GEDI. Relative dissimilarity was categorized for display purposes. Non‐forested areas are in grey. The map projection is EASE‐Grid 2.0 (epsg:6933), a global, equal‐area protection and spatial resolution is 5 km. Map lines delineate study areas and do not necessarily depict accepted national boundaries.

Environmental conditions were well represented (defined here as relative dissimilarity <10%, i.e., ca. a third of maximum dissimilarity) by the system of potential FBRM sites in most lowland tropical rainforests, the eastern part of Canada and the United States of America (USA), northern Europe and the west and central parts of Russia (Figure 1, top). Among forested areas noticeably lacking sufficient coverage of environmental conditions (relative dissimilarity >10%) were the western half of North America (incl. Mexico), Patagonia, Angola/Zambia and eastern Russia. Overall, the geographical and structural spaces benefited from a better representation by the potential FBRM sites than environmental space (Figure 1, centre and bottom, respectively). In the main, only Patagonia, the easternmost part of Siberia and New Zealand were poorly represented in geographical space (relative dissimilarity >10%). Insufficient coverage of structural conditions (relative dissimilarity >10%) mostly affected isolated cells present in limited areas such as the west coast of the USA, forested areas of the Himalayas and the Sunda Shelf (Sumatra, peninsular Malaysia, Borneo) (see Figure S2 for a close‐up on portions of these three areas).

### 3.2 Maximum representativeness possible with different combinations of pre‐existing sites currently identified

**FIGURE 2:**
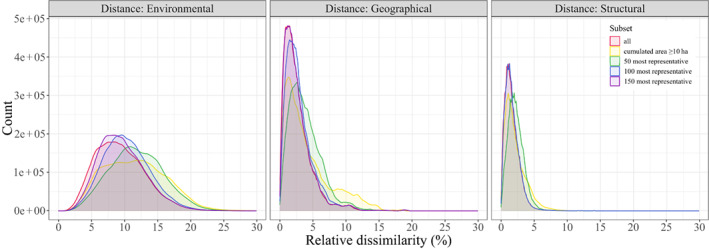
Relative dissimilarities for different types of distances and subsets of potential forest biomass reference measurement sites. There are 1,728,368 contributing cells (5 km spatial resolution) for the environmental (left) and geographical (centre) density plots, and 829,256 for the structural (right) density plot because of GEDI discrete sampling and ISS‐orbit limited spatial coverage (±51.6° latitude). The *X*‐axis was cropped to 30% of relative dissimilarity for display purposes, excluding ca. 0.045% of the overall data.

Comparing the distribution of relative dissimilarity values for environmental, geographical and structural conditions for various sets of potential FBRM sites, the spread (i.e. the variability of values) was highest for environmental space, whatever the set of sites under consideration (Figure 2). Representativeness was always maximized when all the potential FBRM sites were included. Conversely, the highest relative dissimilarity values were reached whatever the space when using the 132 sites with a plot cumulative area ≥10 ha, and not the 50 most representative ones. Consistent with Figure 1, only a low proportion of relative dissimilarity values exceeded 10% when considering geographical or structural conditions. Whatever the value *n*, the identity of the *n* most representative sites differed between spaces, notably because 40% of the sites from the initial pool did not have canopy height information and could therefore not be considered when studying site contribution to the representativeness of the structural space. For a given space, a site among the *n* most representative ones was not necessarily selected for higher values of *n* (Tables S1 and S2; Figure S3).

### 3.3 Pre‐existing site‐ versus random location‐based system

**FIGURE 3:**
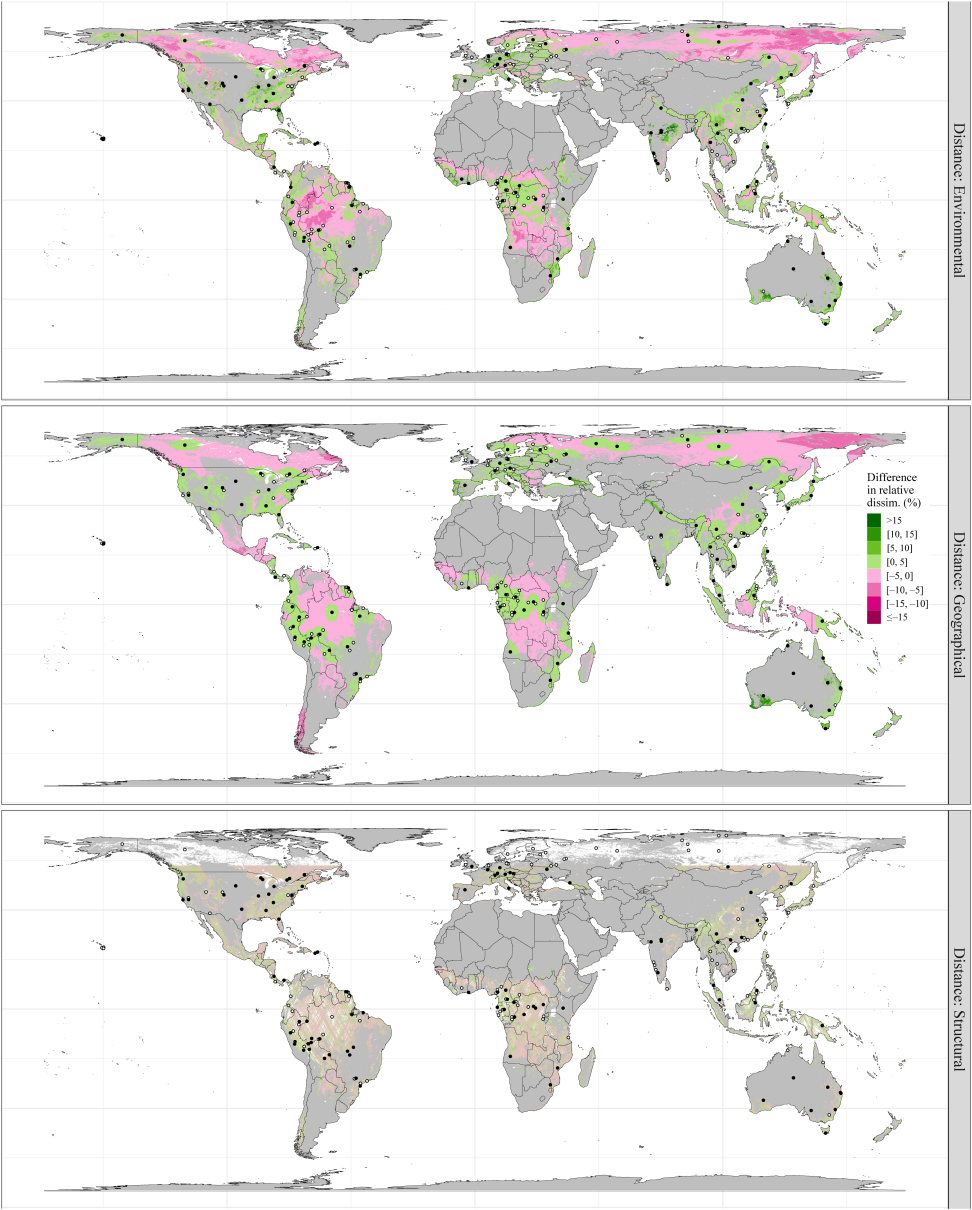
Difference in relative environmental (top), geographical (centre) and structural (bottom) dissimilarities between a set of 100 randomly selected cells (median of 200 runs used) and the 100 most representative potential forest biomass reference measurement (FBRM) sites. A network made up of randomly selected cells is less representative of local conditions than one made up of the 100 most representative potential FBRM sites, wherever the difference in relative dissimilarity is positive. Difference in relative dissimilarity was categorized for display purposes. Non‐forested areas are in grey. Blank continental areas within ±51.6° latitude (bottom) correspond to areas not yet sampled by GEDI, and hollow points to sites not among the 100 most representative potential FBRM sites. The map projection is EASE‐Grid 2.0 (epsg:6933), a global, equal‐area protection and spatial resolution is 5 km. Map lines delineate study areas and do not necessarily depict accepted national boundaries.

Less than half of global forested areas were better represented environmentally, geographically and structurally by the 100 most representative potential FBRM sites than a 100 random samples (proportion ranging from 39% to 48% depending on the space; Figure 3). This was particularly apparent for Canadian, Amazonian, Angolan/Zambian and Russian forested areas with respect to the environmental space (Figure 3, top). Geographically, a better representation was achieved by the 100 most representative potential FBRM sites than by a 100 random ones in the vicinity of selected FBRM sites, creating island‐like patterns (Figure 3, center). Regional patterns were less sharp for the representation of structural conditions, but North American and Asian forested areas appeared generally better represented by the 100 most representative potential FBRM sites than a 100 random samples, while South American and African forested areas showed the opposite (Figure 3, bottom).

### 3.4 Pre‐existing site‐based system improvement

**FIGURE 4:**
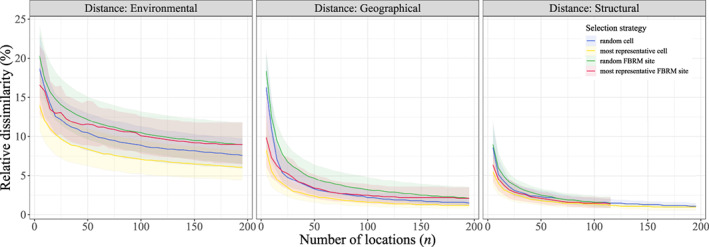
Relative dissimilarities versus number of locations for different types of distances and selection strategies. Only numbers of locations, *n*, which are multiples of 5 are used here. Lines and shaded areas correspond to the median and interquartile range of relative dissimilarity values over global forested areas, respectively.

Increasing plot cumulative area for all sites up to at least 10 ha would increase the number of potential FBRM sites meeting the CEOS requirements (Duncanson et al., 2021), and consequently improve the environmental, geographical and structural coverage of the resulting system (Figure 2). The more locations in the system, the lower the median relative dissimilarity values, whatever the space and location selection strategy (Figure 4). For example, as regards environmental coverage, median relative dissimilarity values decreased from 11.6% to 10.1% to 9.2%, respectively, when the 50, 100 and 150 most representative potential FBRM sites were selected. Selecting the *n* most representative cells over global forested areas always provided better environmental, geographical and structural coverage than other selection strategies. A system made up of random cells was more representative of the environmental and geographical spaces than its most representative pre‐existing site‐based counterpart, whenever at least 20 locations contributed to the system.

## 4 DISCUSSION

### 4.1 Guaranteeing and improving system representativeness

Various ways were identified to guarantee and further improve the representativeness of the proposed system of FBRM sites. First and foremost, efforts (discussed extensively later) should be made to ensure that every single potential FBRM site identified in this study joins the proposed system. The environmental coverage of the system does not seem to improve after at least the 175 most representative potential FBRM sites are included, but geographical and structural coverages showed a continuous although slight improvement (Figure 4).

Second, plot cumulative area should be increased to at least 10 ha at each site wherever this is not the case to comply with CEOS recommendations (Duncanson et al., 2021). This would clearly improve the environmental, geographical and structural coverage of the system (Figure 2), if we were to consider that sites where plots do not cover at least 10 ha overall should consistently be dismissed. Apart from plot cumulative area, ancillary data will likely need to be acquired, updated or upgraded, including more accurate location of plot corners (using differential global navigation satellite systems), soil samples to characterize local soil physicochemical properties, and airborne and terrestrial LiDAR acquisitions. While the FBRM system is being formed, the ‘representativeness of network’ analysis developed in this study can help prioritize sites for main and ancillary data acquisition (Table S2; Figure S3).

Third, efforts should be made to identify pre‐existing sites in areas of poor environmental, geographical or structural coverage (Figures 1 and 3). These include, but are not limited to, Canada, the western half of the USA, Mexico, Patagonia, Angola, Zambia, eastern Russia, tropical and subtropical highlands (e.g. in Colombia, the Himalayas, Borneo, Papua). It should be noted here that in some of these areas, forest inventory data are already collected but with designs suboptimal to have been identified as potential FBRM sites and included in this study. Nonetheless, there will ideally be opportunities to expand on some key locations.

Fourth, given the obvious coverage gaps in these areas, new sites should be established if none already exist. The manifold added values of long‐term permanent plots compared to newly established ones include good knowledge of site history, the availability of ancillary and recensus inventory data, and the fact that plot remeasurement is cheaper than establishment. Yet, relative dissimilarities are minimized whatever the space when most representative virtual sites (i.e. cells) instead of most representative potential FBRM sites are used (Figure 4). This likely arises from the fact that potential FBRM sites are not located randomly. Individual plot networks were usually built with certain criteria in mind, for example, to study well‐defined geographical areas (e.g. Australia for TERN; Cleverly et al., 2019) and/or to answer specific research questions (e.g. what are the long‐term effects of logging on tropical forests for TmFO?; Sist et al., 2015). However, their aggregation does not guarantee a satisfactory representativeness of the environmental, geographical and structural spaces covered by global forested areas. Within a given biome or ecoregion, plot location might also be biased due to, for example, logistical considerations like accessibility. Such could be the case over Amazonia, where a recent study suggested that plots were preferentially located in areas of high ancient human impact, potentially slanting our understanding of Amazonian forest dynamics (McMichael et al., 2017).

Last, to improve the system representativeness and avoid presenting a potentially distorted picture of its performances regionally (e.g. over‐optimistic in the tropics?; see Figure 1), other spaces could be considered, such as biogeographical and disturbance (both exogenous and anthropogenic) spaces. The former could include, among other information, layers of global tree species α and β diversity (Keil & Chase, 2019). The latter could encompass map‐based information on, for example, forest integrity with respect to anthropogenic pressures (Grantham et al., 2020) or susceptibility to natural disturbances (windstorms, wildfires, etc.). Concurrently, integration to the FBRM system of long‐term permanent plot networks focused on the study of secondary forests such as 2ndFOR (Poorter et al., 2021) should be favoured to keep increasing the heterogeneity of forest conditions and successional stages covered by ground data.

### 4.2 Relationship between forest structure and aboveground biomass

Environmental conditions are used to model potential (i.e. theoretical) aboveground biomass (Prentice et al., 2011). Differences between potential and actual biomass stocks are hypothesized to originate from human disturbances (Pan et al., 2013). Structural conditions were represented in this study using remote sensing data (TC fraction and canopy height derived from PROBA‐V and GEDI data, respectively) acquired during the last 2–3 years. Their contemporaneity is an asset to keep track of biomass stocks in a rapidly changing world.

Aboveground biomass is commonly estimated from structural attributes across various scales, using, for example, tree height and diameter at the individual tree scale (Chave et al., 2014) and top‐of‐canopy height at the (sub‐)hectare scale (Labrière et al., 2018). At larger scale, previous exploratory work showed that spatial variations in the product of TC fraction and canopy height closely corresponded to those of LiDAR‐derived aboveground biomass carbon density (AGCD) maps (see ‘CCI Biomass Product Validation and Algorithm Selection Report’ 1 and 2; https://climate.esa.int/en/projects/biomass/key‐documents/). We tested how well TC × *H* correlated with AGCD at the 5 km cell scale over global forested areas and for the subset of cells bearing potential FBRM sites. AGCD estimates were obtained from Spawn et al. (2020), after original data at 300 m spatial resolution were reprojected and averaged to 5 km. We found that AGCD was strongly correlated with TC × *H* over global forested areas (*n* = 829,256, Spearman's *ρ* = .77, *p* < .001) (Figure S4). Root‐mean‐square error (RMSE), coefficient of correlation (*R* ^2^) and bias were 26.7 MgC ha^−1^, 0.85 and 4.1 MgC ha^−1^, respectively. Similar statistics were obtained with potential FBRM site‐bearing cells only (*n* = 118): Spearman's *ρ* = .74 (*p* < .001), RMSE = 30.5 MgC ha^−1^, *R* ^2^ = .82 and bias = 3.5 MgC ha^−1^. This confirmed that structural attributes are important predictors of aboveground biomass.

Nonetheless, local information may be essential to reduce uncertainties in aboveground biomass due to locally variable parameters such as community wood density (Phillips et al., 2019) inferred using tree‐by‐tree identity information that at present can only be provided by in situ data. The pivotal role of in situ data was recently exemplified in the case of GEDI waveform data. Accurately predicting AGCD from GEDI waveforms alone was shown to be suboptimal as two forest stands with similar waveforms can have very different AGCD (Bruening et al., 2021), and allometries heavily rely on in situ training data (Duncanson et al., 2022). Beyond such direct use, tree‐by‐tree identity information can also be mobilized to calibrate and validate hyperspectral data (Draper et al., 2019; Jucker et al., 2018), which can, in turn, improve forest stratification and the use of the most relevant structure metrics‐based allometries. In this study, structural coverage was represented by two of the most meaningful variables that can be remotely sensed over global forested areas: TC fraction and canopy height. Including other structure‐related variables, such as canopy height variability, could complement our understanding of how representative the proposed FBRM sites are of the structural space. This analysis will gain in completeness as new datasets, and new versions of the ones we used, are released. The current GEDI L3 gridded dataset (Version 2) is still patchy, especially in the tropics, and coverage should keep improving with following versions. In addition, plant area index and vertical foliage profile, two variables that have already proven useful to distinguish vegetation types (see e.g. Marselis et al., 2018), should be part of the next releases. Also, as boreal forests are barely sampled by GEDI due to ISS‐orbit limited spatial coverage, incorporating canopy height information from NASA's Ice, Cloud and Land Elevation Satellite‐2 (ICESat‐2) mission (ATL08; Neuenschwander & Pitts, 2019) will help fill a major gap in structure data. As a complement to ICESat‐2, the upcoming NASA‐ISRO's NISAR and ESA's BIOMASS missions will guarantee a continuity in data acquisition for canopy height estimation after the potential end of the GEDI mission (early 2023). Note that at the time of writing NASA was actively exploring options for keeping GEDI on orbit past 2023.

### 4.3 On the uniqueness of tropical forests

The proposed system of FBRM sites should encompass a wide variety of forest conditions (incl. old‐growth, regenerating, managed) and soil types (incl. well‐drained, nutrient‐poor, seasonally flooded, swampy). Adequate coverage of the three main forest biomes (tropical, temperate and boreal) is also essential. But how should this ‘adequate’ coverage be established? Areal forest biome proportions of global forested areas are close to 50%, 20% and 30% for tropical, temperate and boreal forest biomes, respectively (Pan et al., 2013). Based on areal considerations only, this would mean that half of the potential FBRM sites should be located in the tropics, a fifth in temperate regions and the rest (about a third) in boreal ones. This condition is satisfied for most representative virtual sites (i.e. cells), whatever the value *n* of cells and for both environmental and geographical distances, but not for most representative potential FBRM sites (Figure S5). This is likely due to the different balance of forest biome proportions in the list of potential FBRM sites (ca. 60%, 35% and 5% for tropical, temperate and boreal forest biomes, respectively) compared to global forested areas. Regarding structural coverage, forest biome proportions are most probably influenced by the truncated coverage of boreal forests (see above). In terms of aboveground biomass instead of area, forest biome proportions would be 65%, 20%, 15% for tropical, temperate and boreal forest biomes, respectively (using data from Spawn et al., 2020). Focusing on either gross or net primary productivity (GPP and NPP, respectively) also shows the disproportionate contribution of tropical forests compared to their area (more than two‐thirds; Pan et al., 2013), which is even more apparent when emphasizing on gross forest emissions (almost four‐fifths over the years 2001–2019; Harris et al., 2021). Tropical sites should consequently be the cornerstone of the FBRM system, reasonably representing 65%–70% of all the potential FBRM sites. This is all the more relevant because 80%–95% of all known tree species in each continent were sampled in their tropical region (Cazzolla Gatti et al., 2022), a hyperdiversity further complicating community wood density determination (Phillips et al., 2019). While the PAM algorithm does not, either in its original or most recent form, include weighting options, other clustering techniques could be envisioned that would allow weighting existing or virtual potential FBRM sites depending on a cell's AGCD, GPP, NPP, tree diversity or a combination of some or all of these.

### 4.4 Practical implementation of a FBRM system and final considerations

The proposed FBRM system will provide a framework within which a diverse community of stakeholders (e.g. EO agencies, individual countries, forest organizations) can make a lasting contribution to (and of course benefit from) a comprehensive and sustained system of high‐quality biomass reference data. This system also has to be recognized and supported as an opportunity to train the next generation of researchers with expertise at the confluence of forest science and remote sensing, leveraging investments made by the forest science community. Funding the FBRM system will require significant investment. However this investment, even on a global scale, is a fraction of the cost of a single space mission. Plus, this cost is likely to be largely offset by the resulting widespread, consistent and effective use of the EO‐derived biomass maps. Two possible funding mechanisms could be imagined, one where funding bodies collaborate with long‐term permanent plot networks and another where funders collaborate directly with individual plot principal investigators. Whatever the funding scheme favoured, for the FBRM concept to succeed, plot networks must collect and process the data applying the same standards across all countries and continents, and subsequently share the derived data products with the global community, for example through the Forest Observation System (Schepaschenko et al., 2019). Issues on data sharing and data ownership should be limited given that plot networks will not have to share tree‐by‐tree data, only plot/subplot AGCD estimates and associated uncertainties. Protocol harmonization and standardization are key to ensuring high quality of the data generated and maximizing interoperability across all FBRM sites, and should be conducted for all the necessary steps from fieldwork (e.g. plot shape, tree diameter measurement) to post‐field data processing (e.g. allometric equations, error propagation scheme). It must be stressed that the proposed system needs to be established and managed inclusively, with careful consideration of working conditions. Training and site partner involvement in downstream activities should be mandatory. Only this would allow for proper recognition of the disadvantaged social, economic and historical context in which most staff involved in forest research activities operate, which is overwhelmingly true in tropical nations. For further details on the proposed FBRM system, the reader is referred to the GEO‐TREES initiative (Chave et al., 2021).

## CONFLICT OF INTEREST

The authors declare no conflicts of interest.

## Supporting information


Supporting information

Table S1

Table S2

## Data Availability

Land cover data are available from the Climate Data Store (CDS) of the Copernicus Climate Change Service (C3S; https://cds.climate.copernicus.eu/#!/home). Climatic data were obtained from Abatzoglou et al. (2018) (https://doi.org/10.1038/sdata.2017.191). Topographic data were downloaded from the EarthEnv project (http://www.earthenv.org/). Edaphic data are available from SoilGrids 2.0 (https://doi.org/10.5194/soil‐7‐217‐2021). Canopy height information was obtained from the GEDI L3 Gridded Land Surface Metrics, Version 2 dataset (https://doi.org/10.3334/ORNLDAAC/1952). Tree cover fraction data were obtained from Version 3.0.1 of the global land cover maps distributed by the Copernicus Global Land Service (https://doi.org/10.3390/rs12061044). Realm borders are available from Dinerstein et al. (2017) (https://doi.org/10.1093/biosci/bix014). Aboveground carbon density (AGCD) estimates were obtained from Spawn et al. (2020) (https://doi.org/10.1038/s41597‐020‐0444‐4).
