# Supplementary material for: Toward a forest biomass reference measurement system for remote sensing applications
Source: Glob Chang Biol. 2022 Nov 8;29(3):827–40. doi: 10.1111/gcb.16497 (PMC10099565; doi:10.1111/gcb.16497)
Supplement: Supplementary file 1 — Supporting information [file GCB-29-827-s001.pdf]

<u>Supplementary information for “Toward a forest biomass reference measurement system for remote sensing applications”</u>

Study area

The forest mask was built using land cover data for 2020 from the ESA CCI Land Cover project. Original data were downloaded from the Climate Data Store (CDS) of the Copernicus Climate Change Service (C3S; https://cds.climate.copernicus.eu/#!/home). The entire dataset is called "Land cover classification gridded maps from 1992 to present derived from satellite observations", and data can be downloaded on a yearly basis. To build the forest mask, only the following classes were retained: "Tree cover, broadleaved, evergreen, closed to open (>15%)" (class **50**), "Tree cover, broadleaved, deciduous, closed to open (>15%)" (class **60**), "Tree cover, broadleaved, deciduous, closed (>40%)" (class **61**), "Tree cover, broadleaved, deciduous, open (15-40%)" (class **62**), "Tree cover, needleleaved, evergreen, closed to open (>15%)" (class **70**), "Tree cover, needleleaved, evergreen, closed (>40%)" (class **71**), "Tree cover, needleleaved, evergreen, open (15-40%)" (class **72**), "Tree cover, needleleaved, deciduous, closed to open (>15%)" (class **80**), "Tree cover, needleleaved, deciduous, closed (>40%)" (class **81**), "Tree cover, needleleaved, deciduous, open (15-40%)" (class **82**), "Tree cover, mixed leaf type (broadleaved and needleleaved)" (class **90**), "Tree cover, flooded, fresh or brakish water" (class **160**), and "Tree cover, flooded, saline water" (class **170**).

Maximum environmental, geographical and structural distances

We computed Euclidean distances on ten environmental and two structural variables. Considering for each set of variables minimum and maximum values reached over global forested areas would allow to compute theoretical extreme distances. For structural variables, i.e. canopy height and tree cover fraction, while it is plausible that a single cell has maxima for both variables and another cell has both minima, this is unlikely for environmental variables where multiple climatic, topographic and edaphic

variables are under consideration. Therefore, we searched for realized extreme, i.e. minimum and maximum, distances instead of theoretical ones. First, we selected 1,000 cells geographically spanning the study area (different sampling was used for the search of environmental and structural extremes, as the pool of cells was different given GEDI discrete sampling and ISS-orbit limited spatial coverage). Second, for each of those we computed their distance from each other cell over global forested areas, and retained extreme values along with the cell for which each extreme was obtained. Third, we built occurrence tables of those cells involved in extreme distances (4 occurrence tables in total, for minimum/maximum values for environmental/structural conditions). Last, we repeated step 2 for the 15 most common cells identified in each occurrence table and final realized extreme distances were identified consequently. For geographical space, minimum and maximum distances were set to 0 km and half a great circle (ca. 20,037.51 km), respectively.

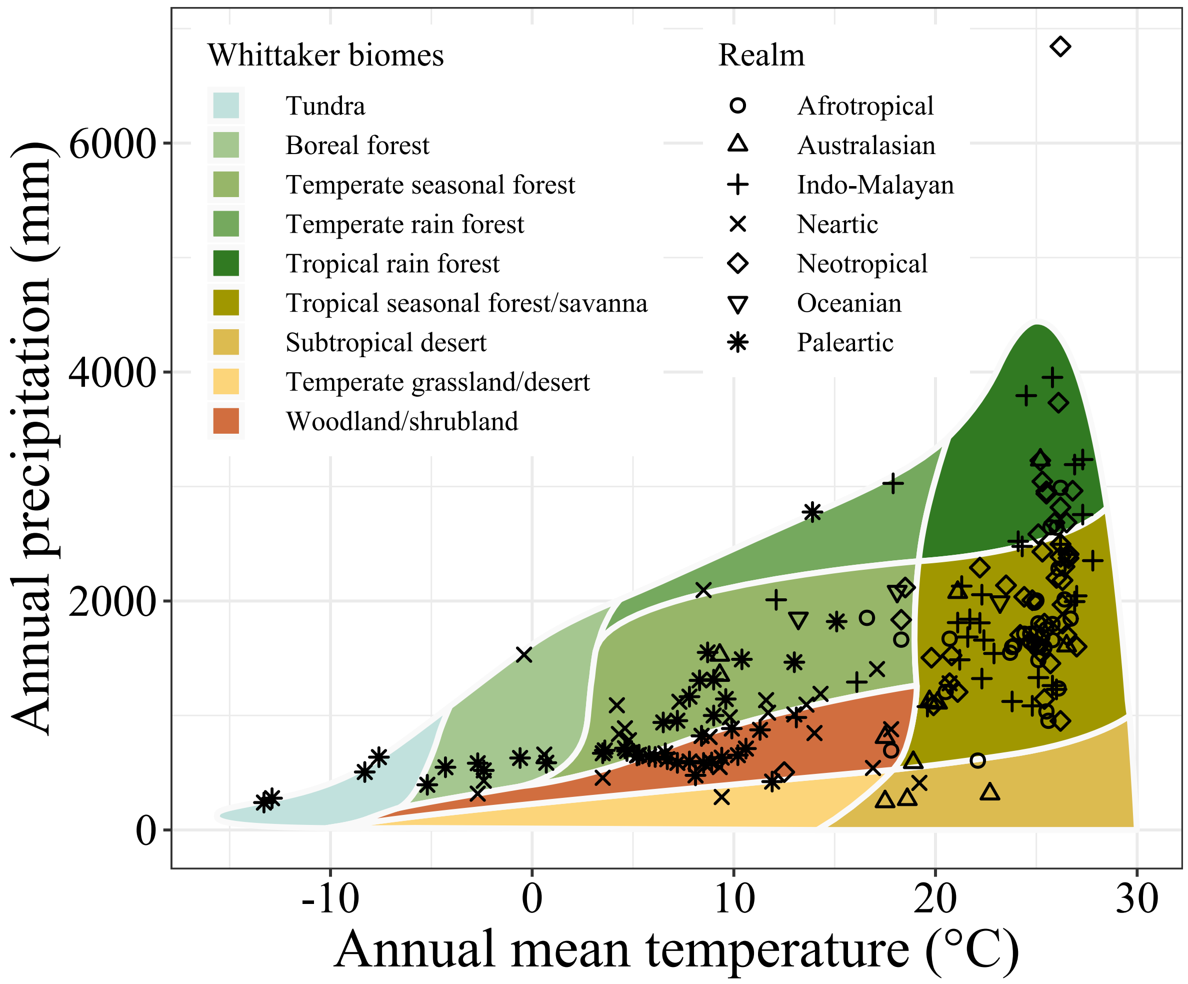

Figure S1. Whittaker diagram showing biome classification and delineation *sensu* Whittaker (1975) as a function of annual precipitation and mean temperature. All the forest biomass reference measurement sites (n = 195) are also displayed, along with information on their respective realm (realm borders obtained from Dinerstein et al., 2017). Note that one potential forest biomass reference measurement site is located in the Colombian part of the “Chocó–Darién moist forests” ecoregion, one of the wettest regions of Earth (annual precipitation > 6,000 mm).

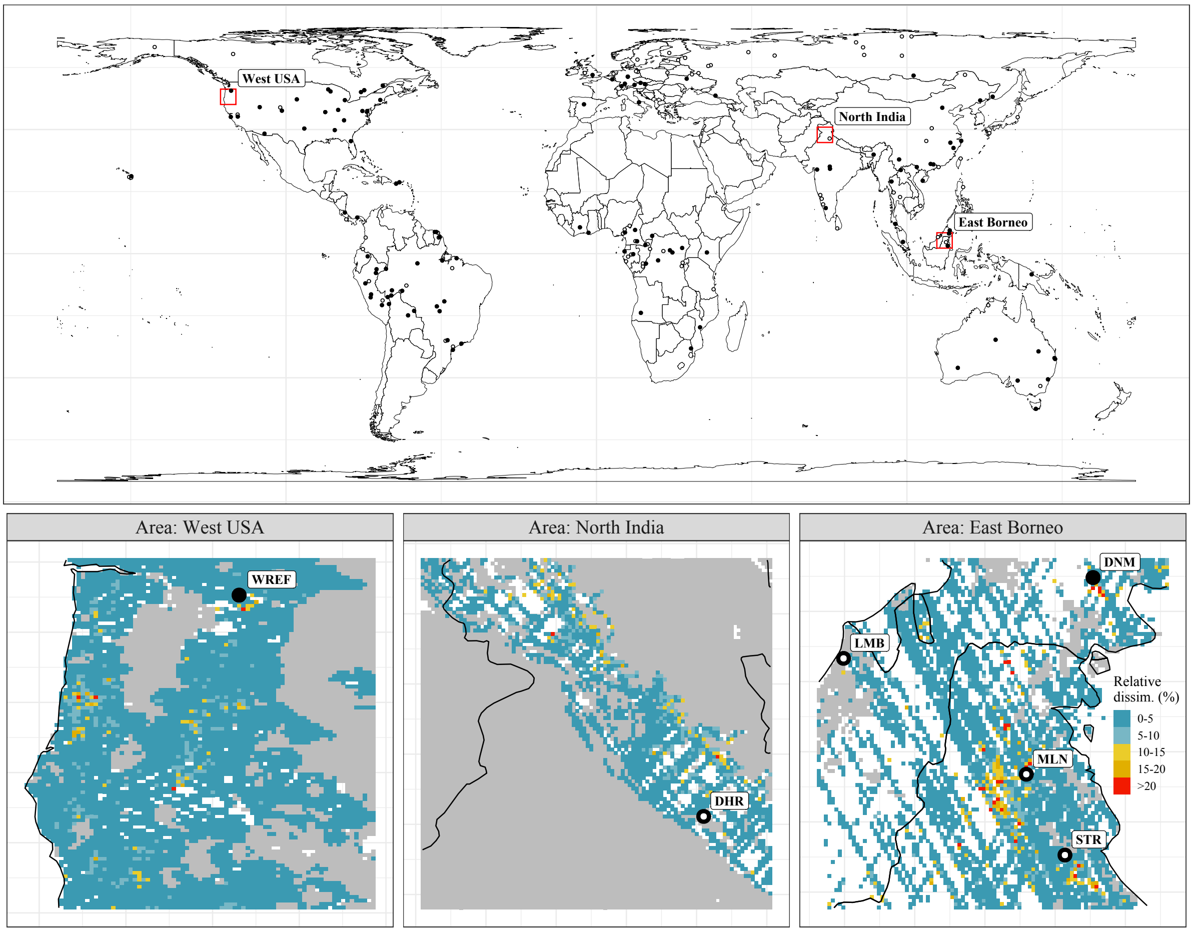

Figure S2. Location (top) and relative structural dissimilarity (bottom) for a subset of forested areas with insufficient coverage (relative dissimilarity > 10%, bottom) with respect to conditions covered by potential forest biomass reference measurement (FBRM) sites. Blank continental areas and hollow points (bottom) respectively correspond to forested areas and sites not sampled (yet) by GEDI, and each facet displays a 500 × 500 km area. Relative dissimilarity was categorized for display purposes. Non-forested areas are in grey. The map projection is EASE-Grid 2.0 (epsg:6933), a global, equal-area protection, and spatial resolution is 5 km.

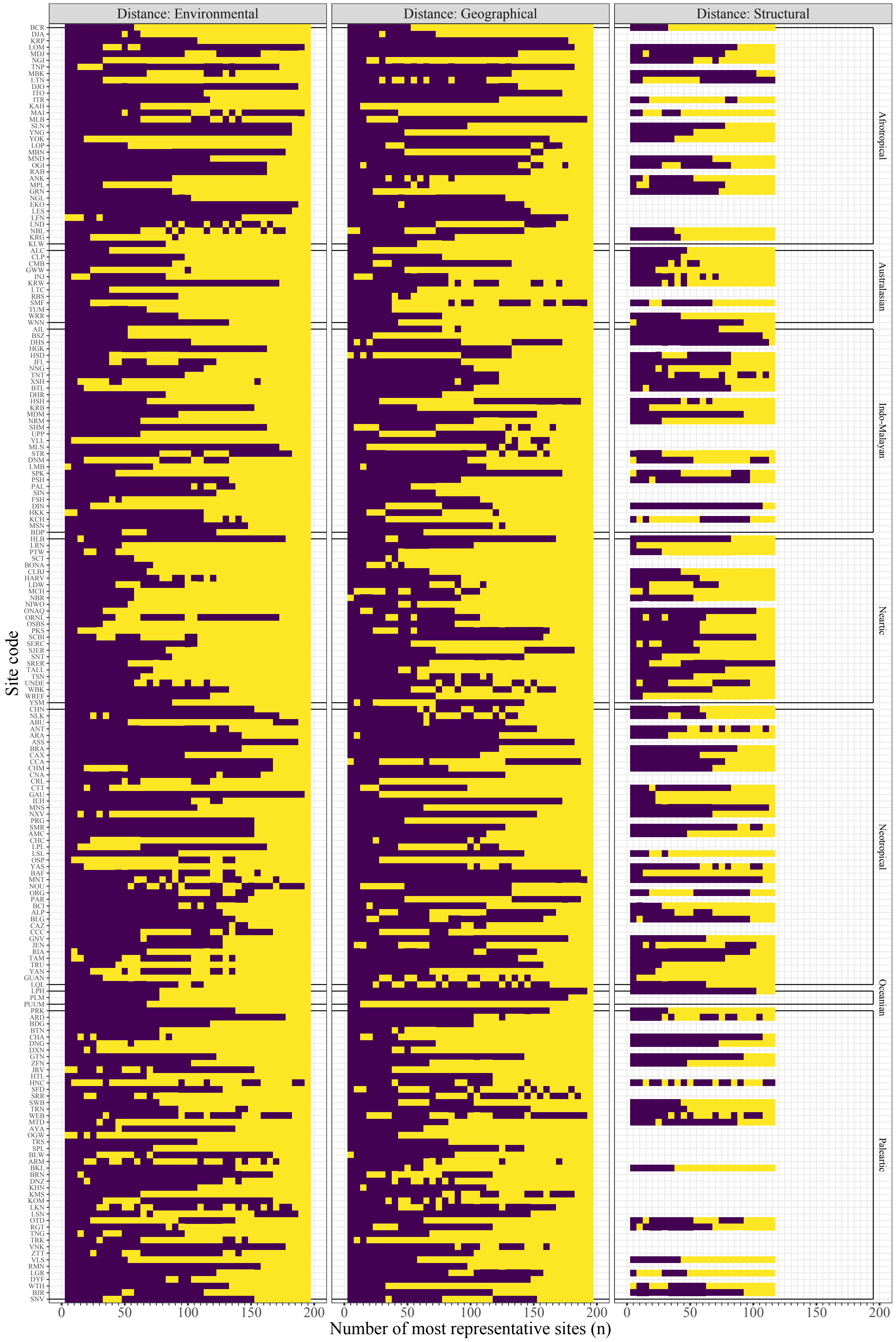
Distance: Environmental
Distance: Geographical
Distance: Structural
Site code
Number of most representative sites (n)
0
50
100
150
200
Afrotropical
Australasian
Indo-Malayan
Nearctic
Neotropical
Oceanian
Palearctic

Figure S3. Most representative potential forest biomass reference measurement sites for different types of distances. Selected and non-selected sites are in light and dark colors, respectively. For display purposes, only numbers, n, of most representative sites that are multiples of 5 are used here. Sites are ordered by biome (biome borders obtained from Dinerstein et al., 2017). The correspondence between site codes and names can be found in Table S1.

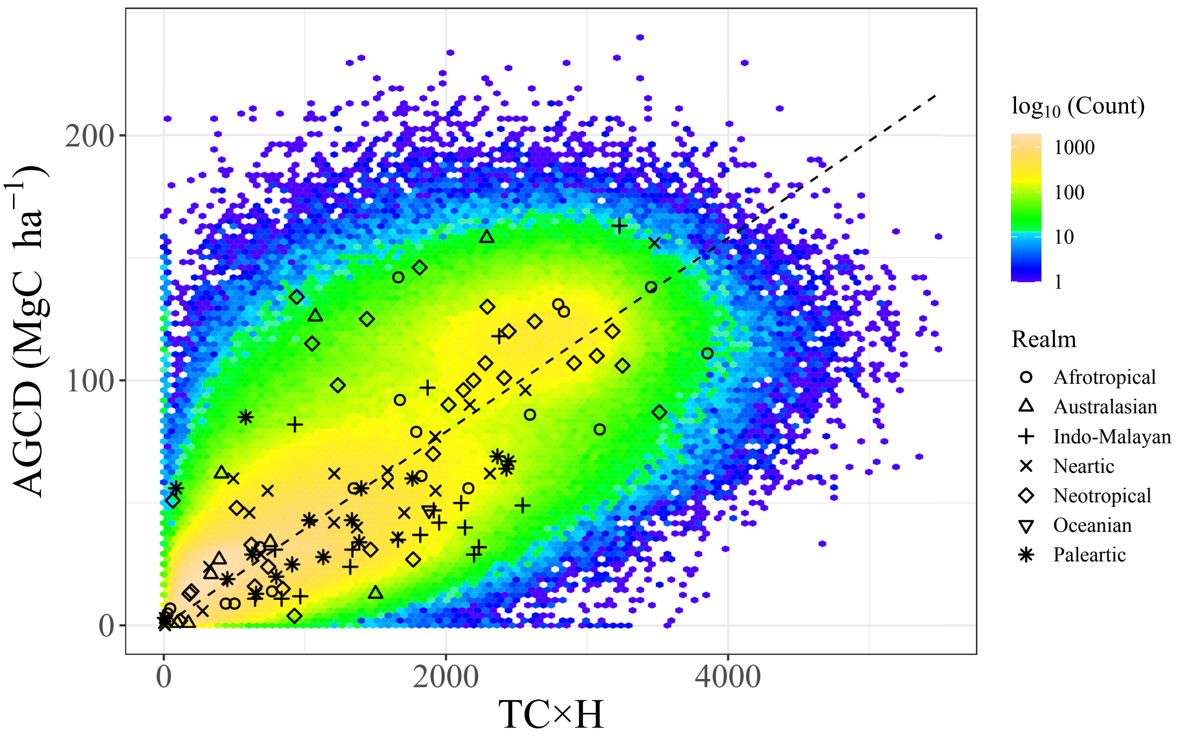

Figure S4. Aboveground biomass carbon density (AGCD; MgC ha^{-1}) vs. tree cover fraction (TC; %) times canopy height (H; m). AGCD estimates were obtained from Spawn et al. (2020). TC and H were derived from Proba-V and GEDI observations, respectively. Data from the 829,256 contributing cells (5 km spatial resolution) were binned together for display purposes (100 bins on both axes). The dashed line represents a linear regression forced through zero. All the potential biomass reference measurement sites with GEDI information (n = 118) are also displayed, along with information on their respective realm (realm borders obtained from Dinerstein et al., 2017).

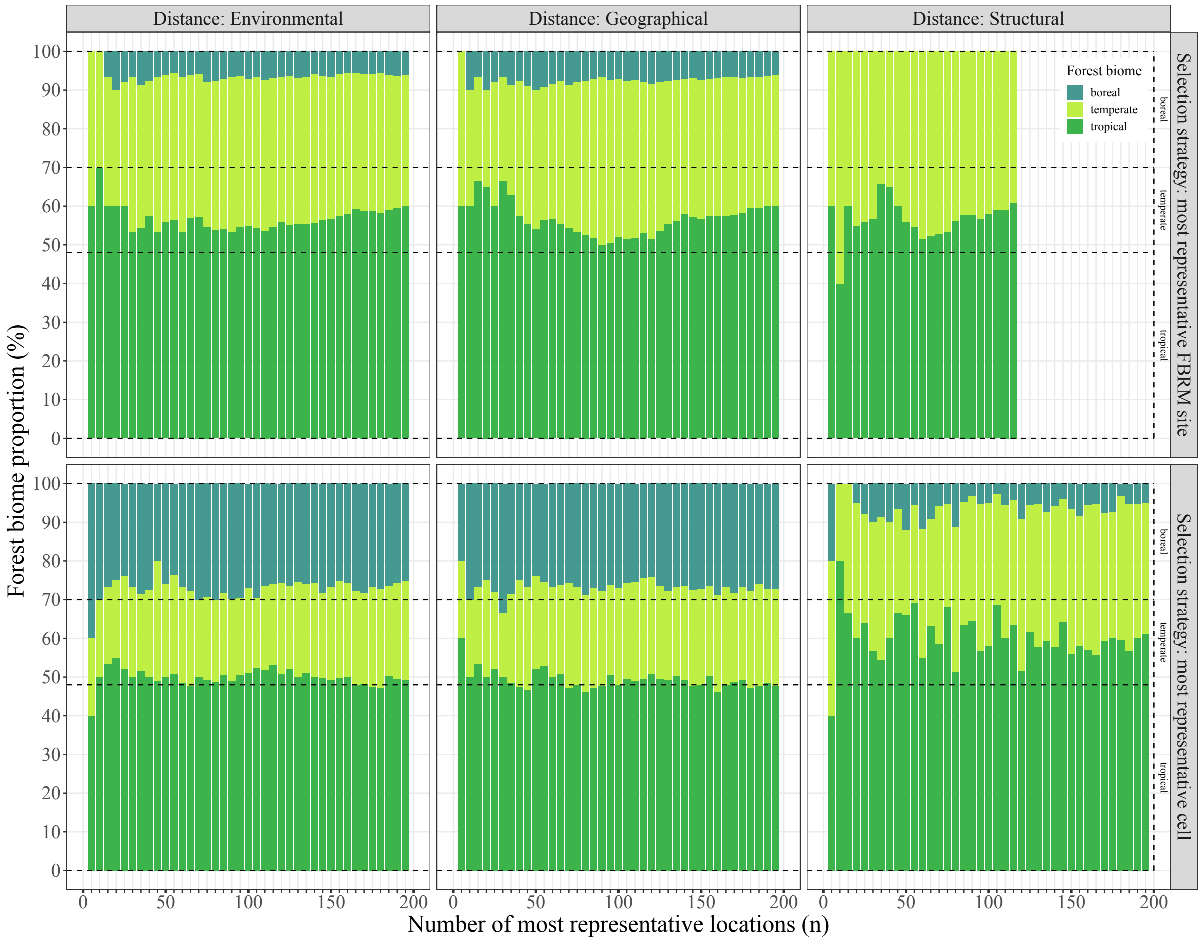

Figure S5. Forest biome proportion (%) vs. number of most representative locations for different types of distances and selection strategies. For display purposes, only numbers n of most representative locations multiple of 5 were used here. Forest biomes *sensu* Dinerstein et al. (2017) were classified as tropical, temperate or boreal. Dashed lines indicate areal forest biome proportions of global forested areas (48, 22 and 30% for tropical, temperate and boreal forest biomes, respectively).

Table S1. Potential forest biomass reference measurement site coordinator, location, plot cumulative area, structural and environmental attributes, and inclusion (+) or not (-) in the set of the 150, 100 and 50 most representative sites. Attribute values were extracted at each site location from the corresponding layers produced at 5 km spatial resolution. Cum. area = plot cumulative area (ha), Year = established year of (first) plot establishment at the site (yr), H = canopy height (m), TC = tree cover fraction (%), AGCD = aboveground biomass carbon density (MgC ha^{-1}; [range]), AMT = annual mean temperature (°C), TSE = temperature seasonality (% coefficient of variation CV), APR = annual precipitation (mm), PSE = precipitation seasonality (% CV), SRAD = downward surface shortwave radiation (W m^{-2}), Elevation = elevation above sea level (m), CFVO = coarse fragments (% volume), Sand = sand fraction (% mass), CEC = cation exchange capacity (cmol kg^{-1}), pH (in H_2O) (unitless).

Table S2. Partitioning of potential forest biomass reference measurement sites depending on number of sites, n, and space (environmental, geographical, structural). Only numbers of sites which are multiples of 5 are used here. Codes correspond to partition medoids. For a given column, all rows with the same code belong to the same partition.

References

Dinerstein, E., Olson, D., Joshi, A., Vynne, C., Burgess, N. D., Wikramanayake, E., Hahn, N., Palminteri, S., Hedao, P., Noss, R., Hansen, M., Locke, H., Ellis, E. C., Jones, B., Barber, C. V., Hayes, R., Kormos, C., Martin, V., Crist, E., … Saleem, M. (2017). An Ecoregion-Based Approach to Protecting Half the Terrestrial Realm. *BioScience*, *67*(6), 534–545. https://doi.org/10.1093/biosci/bix014

Spawn, S. A., Sullivan, C. C., Lark, T. J., & Gibbs, H. K. (2020). Harmonized global maps of above and belowground biomass carbon density in the year 2010. *Scientific Data*, *7*(1), 112. https://doi.org/10.1038/s41597-020-0444-4

Whittaker, R. H. (1975). *Communities and Ecosystems*. Macmillan.
